# Structural and Functional Characterization of Fibronectin in Extracellular Vesicles From Hepatocytes

**DOI:** 10.3389/fcell.2021.640667

**Published:** 2021-03-18

**Authors:** Xinlei Li, Ruju Chen, Sherri Kemper, David R. Brigstock

**Affiliations:** ^1^Center for Clinical and Translational Research, Abigail Wexner Research Institute at Nationwide Children’s Hospital, Columbus, OH, United States; ^2^Department of Surgery, The Ohio State University Wexner Medical Center, Columbus, OH, United States

**Keywords:** extracellular vesicle, exosome, fibronectin, integrins, receptor, endocytosis

## Abstract

Extracellular vesicles (EVs) are membrane-limited nanoparticles that are liberated by cells and contain a complex molecular payload comprising proteins, microRNA, RNAs, and lipids. EVs may be taken up by other cells resulting in their phenotypic or functional reprogramming. In the liver, EVs produced by non-injured hepatocytes are involved in the maintenance of hepatic homeostasis or therapeutic outcomes following injury while EVs produced by damaged hepatocytes may drive or exacerbate liver injury. In this study, we examined the contribution of EV fibronectin (FN1) to the biogenesis, release, uptake, and action of hepatocyte-derived EVs. While FN1 is classically viewed as a component of the extracellular matrix that regulates processes such as cell adhesion, differentiation, and wound healing and can exist in cell-associated or soluble plasma forms, we report that FN1 is also a constituent of hepatocyte EVs that functions in EV uptake by target cells such as hepatocytes and hepatic stellate cells (HSC). FN1 co-purified with EVs when EVs were enriched from conditioned medium of human or mouse hepatocytes and a direct association between FN1 and hepatocyte EVs was established by immunoprecipitation and proteinase protection. FN1 ablation in mouse hepatocytes using CRISPR-Cas9 did not alter EV biogenesis but EV uptake by HSC was significantly reduced for FN1 knockout EVs (EV^Δ*FN1*^) as compared to EVs from wild type hepatocytes (EV^WT^). The uptake by hepatocytes or HSC of either EV^WT^ or EV^Δ*FN1*^ required clathrin- and caveolin-mediated endocytosis, cholesterol, lysosomal acidic lipase activity, and low pH, while macropinocytosis was also involved in EV^Δ*FN1*^ uptake in HSC. Despite their differences in rate and mechanisms of uptake, EV^Δ*FN1*^ functioned comparably to EV^WT^ in ameliorating CCl_4_-induced hepatic fibrosis in mice. In conclusion, FN1 is a constituent of hepatocyte EVs that facilitates EV uptake by target cells but is dispensable for EV-mediated anti-fibrotic activity *in vivo*.

## Introduction

Extracellular vesicles (EV) are membrane-limited nanoparticles that are released by virtually all cell types and which mediate intercellular communication through the delivery to target cells of complex molecular payloads (van Balkom et al., 2015; Maas et al., 2017). In the liver, EV-mediated communication between hepatocytes and non-parenchymal cells such as hepatic stellate cells (HSC), liver sinusoid endothelial cells, Kupffer cells, or infiltrating immune cells contributes to the regulation of normal physiological functions, homeostasis or pathogenesis (Racanelli and Rehermann, 2006; Kubes and Jenne, 2018). The manner in which hepatocyte EVs regulate these processes is highly dependent on the phenotypic status of the donor hepatocytes. On the one hand, a pro-pathogenic role for hepatocyte EVs is demonstrated by the findings that cultured hepatocytes exposed to free fatty acids to mimic non-alcoholic fatty liver disease (NAFLD)-like lipotoxicity produce EVs that contain non-alcoholic steatohepatitis (NASH)- or fibrosis-related inflammatory cargos which stimulate proinflammatory cytokine production in macrophages or fibrogenesis in HSC (Povero et al., 2015; Hirsova et al., 2016; Lee et al., 2017; Dasgupta et al., 2020). Similarly, in animal models of alcoholic liver disease, CD40 ligand-enriched hepatocyte EVs drive macrophage activation and inflammatory cytokine production (Verma et al., 2016). In acute liver injury and early fibrosis, hepatocyte EVs induce toll-like receptor 3 expression in HSC, leading to HSC activation that is perpetuated by an interleukin-17 positive feedback mechanism between HSC and γδ-T cells (Seo et al., 2016), while hepatocytes infected with hepatitis C virus produce EVs that are enriched in microRNAs that drive fibrogenic gene expression in HSC (Devhare et al., 2017; Kim et al., 2019). Moreover, hepatoma-derived EVs contribute to liver tumor progression by inhibiting tumor-suppressor genes, increasing vesicular permeability, generating a pro-metastatic microenvironment, or weakening immune surveillance (Zhang et al., 2017; Fang et al., 2018; Xue et al., 2018; Han et al., 2019). On the other hand, EVs from normal hepatocytes have beneficial or therapeutic effects, including stimulation of hepatocyte proliferation and liver regeneration in partial hepatectomy or ischemia reperfusion injury models (Nojima et al., 2016) as well as anti-fibrotic actions in experimental liver fibrosis (Li et al., 2019). T he therapeutic properties of hepatocyte EVs involved promoting hepatocyte repair and suppressing fibrotic gene expression in HSC downstream of their binding to cell surface heparin-like molecules or integrins (Chen and Brigstock, 2016; Chen et al., 2018; Li et al., 2019). These receptors were also shown to mediate binding and internalization of EVs by glioblastoma or myeloma cells through their interactions with EV-associated fibronectin (FN1) (Christianson et al., 2013; Purushothaman et al., 2016).

FN1 is traditionally viewed as existing in soluble plasma or cell-associated forms that are produced, respectively, by hepatocytes or many cell types (Tamkun and Hynes, 1983; Christiansen et al., 1988; Sakai et al., 2001; Pankov and Yamada, 2002; Klemis et al., 2017). Cell-associated FN1 contains two alternatively spliced repeats, EDA and EDB, that are absent from plasma FN1 (To and Midwood, 2011) even though both types of FN1 have many functional similarities. Through its binding to cell membrane receptors comprising heparan sulfate proteoglycans (HSPG) or integrins (e.g., α5β1), FN1 regulates diverse cellular processes such as adhesion, migration, and proliferation (Richardson et al., 2001; Zhan et al., 2009; Hamidi and Ivaska, 2017). FN1 turnover involves caveolin-mediated endocytosis and lysosomal entry (Sottile and Chandler, 2005; Shi and Sottile, 2008, 2011). However, the compartmentalization of FN1 into EVs is emerging as a novel and consistent feature of several cell types including HSC, melanocytes, trophoblasts, or cancer cells but with few exceptions mechanistic and functional studies are lacking (Antonyak et al., 2011; Atay et al., 2011; Peinado et al., 2012; Sung et al., 2015; Bin et al., 2016; Purushothaman et al., 2016; Li et al., 2020). Since FN1 has not been previously studied in the context of hepatocyte EVs, we have analyzed the role of FN1 in EV biogenesis, release, cell binding and internalization, and action. By generating EVs that are null for FN1 and comparing them to wild type EVs, we show that hepatocyte EV production and action are FN1-independent but that FN1 regulates aspects of EV binding to and internalization in target hepatocytes or HSC.

## Materials and Methods

### Cells

The wild type (WT) mouse hepatocyte line, AML12 (CRL-2254, American Type Culture Collection (ATCC, Manassas, VA, United States), and its derivatives were cultured in DMEM/F12 medium (Thermo Fisher Scientific, Waltham, MA, United States) supplemented with 10% fetal bovine serum (FBS; Corning Inc., Corning, NY, United States), and 1% penicillin-streptomycin-antifungal containing insulin, transferrin, selenium and dexamethasone (Lonza, Alpharetta, GA, United States). Human hepatocyte HepG2 cells (HB-8065; ATCC) or HEK293T cells (CRL-3216; ATCC) were cultured in DMEM containing 10% FBS. Primary mouse hepatocytes or mouse hepatic stellate cells (mHSC) were isolated from male wild-type Swiss Webster mice (6–8 weeks) by perfusion and digestion of livers, followed by buoyant-density centrifugation as previously described (Li et al., 2019). Animal procedures were approved under protocol #04504AR by the Institutional Animal Use and Care Committee at Nationwide Children’s Hospital (Columbus, OH, United States). Mouse HSC were verified for purity, identity and phenotypic transition into activated pro-fibrogenic myofibrobalsts over the first week in culture as described (Li et al., 2020) and the cells were maintained in DMEM/F12/10% FBS/1% penicillin-streptomycin-antifungal, and split when confluent for use up to passage 6 (P6).

### Generation of Knock-out or Knock-down Cells

The knock-out oligonucleotides containing the target sequence of guide RNA (gRNA) in the mouse fibronectin (mFN1) genome (GAC TGT ACC TGC ATC GGG GC) were annealed and inserted into lentiviral vector lentiCRISPR-V2 (plasmid # 52961; Addgene, Watertown, MA, United States), which was a gift from Dr. Feng Zhang (Massachusetts Institute of Technology, Cambridge, MA, United States) (Sanjana et al., 2014). The lentiCRISPR-V2-mFN1 gRNA was confirmed by sequencing. Lentiviruses were produced by transfecting HEK293T cells with original lentiCRISPR-V2 or lentiCRISPR-V2-mFN1 gRNA, psPAX2, and pCMV-VSVG at a ratio of 3:2:1, and collecting culture supernatants at 48 and 72 h post-transfection. The supernatants were briefly centrifuged at 1,000 × *g* for 10 min, clarified by passage through a 0.45 μm filter, and used to transduce AML12 cells which were cultured under selection with 2 μg/ml puromycin (InvivoGen, San Diego, CA, United States). The lentiCRISPR-V2-transduced cells were used as scramble control cells. The positive cell population was collected and subjected to single clone selection. The knock-out cells were validated by immunostaining of mFN1 and genome sequencing. Two positive mFN1 knockout clones, hereafter referred to as ΔFN1 cells, were randomly selected for the experiments.

The knock-down oligonucleotides containing the target sequence of clathrin-1 heavy chain (CLTC, GATTACCAAGTATGGTTATAT), caveolin-1 (CAV1, CGACGTGGTCAAGATTGACTT), or Dynamin-2 (DNM2, GCCCTTGAGAAGAGGCTATAT) were annealed and inserted into lentiviral vector pLKO.1, a kind gift from Dr. Zongdi Feng (Nationwide Children’s Hospital, Columbus, OH, United States). The insertions were justified by restriction enzyme digestion and sequencing. The lentiviral stocks were generated as described above and used to transduce AML12 cells or passaged mHSC. The positive cell population was selected with puromycin and the knock-down efficiency was confirmed by Western blot. The pLKO.1-transduced cells were used as scramble control cells.

### Hepatocyte EV Purification

Mouse or human hepatocytes were plated in T175 flasks until they reached >90% confluency after which spent medium was removed, and the cells were rinsed twice with Hanks Balanced Salt Solution (Thermo Fisher, Waltham, MA, United States) prior to incubating with serum-free medium overnight, followed by replacement with fresh serum-free medium for 48 h. The supernatants were subjected to sequential centrifugation (300 × *g* for 10 min, 2,000 × *g* for 20 min, 10,000 × *g* for 30 min) and ultracentrifugation (100,000 × *g* for 70 min at 4°C) in a Type T70i fixed-angle rotor, the pellet from which was resuspended and subjected to the same ultracentrifugation conditions again. The resulting EV pellet was dispersed in PBS and characterized as described below. EVs from wild type or ΔFN1 AML12 cells are hereafter named “EV^WT^” or “EV^Δ*FN1*^,” respectively. For some experiments, serum used for tissue culture was depleted of its constituent EVs by ultracentrifugation at 100, 000 × *g* for overnight (Thery et al., 2018).

Extracellular vesicles were labeled either by membrane staining using PKH26 (MilliporeSigma, St. Louis, MO, United States) as described (Li et al., 2019) or by labeling of their RNA payload by incubation of AML12 producer cells with RNAselect (Thermo Fisher Scientific, Waltham, MA, United States).

### Extracellular Vesicles Characterization

Extracellular vesicles were subjected to Nanoparticle Tracking Analysis (NTA) using a Nanosight 300 (Malvern Instruments, Westborough, MA, United States) that had been calibrated with 100 nm polystyrene latex microspheres. Recordings were performed at room temperature with a camera gain of 15 and a shutter speed of 4.13 ms. The detection threshold was set to 6. Each EV sample was analyzed twice for determination of mean particle concentration and size distribution.

An enzyme-linked immunosorbent assay (ELISA) was used to quantify FN1 in culture supernatant, large vesicle pellets recovered from 10,000 × *g* centrifugation, and EV pellets after 100,000 × *g* ultracentrifugation, using a commercial kit (cat# LS-F2426, LifeSpan BioSciences, Seattle, WA, United States).

A proteinase K digestion protection assay was performed by treating 20 μg of EV^WT^ with or without 1% NP40 at 37°C for 15 min, followed by incubation with proteinase K (0, 20, 200 μg/ml) for another 30 min. FN1 and flotillin-1 were detected by Western blot in control or digested samples.

### Western Blot

Western blot was used to detect common EV marker proteins and FN1. 10–20 μg protein from EV or cell lysates were subjected to sodium dodecyl polyacrylamide gel electrophoresis (SDS-PAGE). Blots were incubated with primary antibodies to CD63 (1:100; MilliporeSigma, Burlington, MA, United States), flotillin-1 (1:200; BD Biosciences, San Jose, CA, United States), CD9 (1:500; Abcam, Cambridge, MA, United States), HNF4α (1:200, Thermo Fisher Scientific, Waltham, MA, United States), clusterin (Clu, 1:500; Proteintech, Rosemont, IL, United States), major vault protein (MVP; 1:500; Proteintech, Rosemont, IL, United States), albumin (1:500, Abcam, United States), mFN1 (1:500, Abcam, United States), huFN1 (1:500, Sinobiological, Beijing, China), or β-actin (1:1,000; Invitrogen, Carlsbad, CA, United States). Blots were developed using an Odyssey Imaging System (LI-COR Biosciences, Lincoln, NE, United States). CLTC (ab21679, Abcam), CAV1 (ab2910, Abcam), and DNM2 (A303-513A, Bethyl Laboratories, Montgomery, TX, United States) antibodies were used to measure the knockdown efficiency of their respective targets in AML12 or passaged mHSC transduced with lentiviral short-hairpin RNA.

### Extracellular vesicles Protein Digestion and Mass Spectrometry

The purified EV samples were subjected to digestion and mass spectrometry as described previously (Li et al., 2020). Briefly, EV pellets were resuspended in 50 mM ammonium bicarbonate containing 0.1% Rapigest (Waters Corp., Milford, MA, United States), homogenized by sonication, and clarified by centrifugation at 13,000 rpm. Protein concentration was determined using a Qubit assay kit (Thermo Fisher Scientific, Waltham, MA, United States), dithiothreitol and iodoacetamide were sequentially added before sequencing grade trypsin (Promega Corp., Madison, WI, United States) was added for digestion for overnight at 37°C. Trifluoroacetic acid was then added to precipitate the Rapigest which was then removed by centrifugation. The clarified supernatant was dried and resuspended in 20 μl 50 mM acetic acid. Peptide concentration was determined at 280 nm using a nanodrop spectrophotometer (Thermo Fisher Scientific, Waltham, MA, United States). Three separate EV^WT^ preparations were individually prepared for mass spectrometry.

Extracellular vesicles protein identification was performed using nano-liquid chromatography-nanospray tandem mass spectrometry (LC/MS/MS) on a Thermo Scientific Q Exactive mass spectrometer equipped with an EASY-Spray^TM^ Sources operated in positive ion mode. The MS/MS analysis was programmed for a full scan recorded between m/z 400–1600 and an MS/MS scan to generate product ion spectra to determine amino acid sequence in consecutive scans starting from the most abundant peaks in the spectrum, and then selecting the next nine most abundant peaks.

Sequence information from the MS/MS data was processed by converting the raw files into a merged file using MS convert (ProteoWizard). The resulting mgf files were searched using Mascot Daemon by Matrix Science version 2.6.0 (Boston, MA, United States) and the database searched against Uniprot Mouse database. A decoy database was also searched to determine the false discovery rate (FDR) and peptides were filtered according to the FDR. Proteins with less than 1% FDR as well as a minimal of two significant peptides detected were considered as valid proteins. Proteomics data were summarized in Scaffold 4.9.0 (Proteome Software Inc., Portland, OR, United States) for spectral counting analysis. Complete MS datasets are available in the Supplemental Data.

### Gene Ontology, Pathway Enrichment, and Protein-Protein Interaction Networks

Gene Ontology (GO^1^ and the Kyoto Encyclopedia of Genes and Genomes (KEGG^2^) analyses of EV proteins were accomplished using the DAVID online program^3^. Search Tool for the Retrieval of Interacting Genes (STRING^4^ was utilized to determine interactions among EV proteins using a medium interaction score of 0.7, and the Markov Cluster Algorithm method with an inflation parameter of 3 was applied for clustering. These analyses were each performed with a criterion FDR < 0.05.

### Immunoprecipitation (IP)

100 μg of purified AML12 cell EVs were incubated with 4 μg of anti-mFN1 antibody (Abcam) or with 4 μg isotype normal IgG at room temperature for 3 h, followed by addition of 50 μl of Dynabeads (cat#10004D, Thermo Fisher Scientific, Waltham, MA, United States) and incubation overnight at 4°C. Beads were then magnetically separated and the unbound material was retained. The beads were washed with PBS-0.02% Tween four times before resuspending them in PBS. The immunoprecipitated samples and the unbound material were boiled and subjected to SDS-PAGE under reducing conditions, followed by Western blot. CD9, flotillin-1, and FN1 primary antibodies and IP-specific secondary antibodies (ab121366 VeriBlot HRP secondary IgG, ab131368 rat anti-mouse IgG, Abcam) were used for detection.

### EV Uptake in mHSC or Hepatocytes

Mouse HSC or hepatocytes seeded in 96-well plates were cultured in, respectively, 2% exosome-depleted serum-containing medium or serum-free medium overnight. The following day, the plates were placed on ice and cells were treated with PKH26- or RNASelect-labeled EVs in the presence or absence of a panel of endocytosis or macropinocytosis inhibitors to assess uptake pathways for EV internalization. These included clathrin-mediated endocytosis (ClME) inhibitor chlorpromazine (CPZ, cat# HY-B0407A, MCE; Monmouth Junction, NJ, United States); caveolin-mediated endocytosis (CaME) inhibitors including Genistein (cat# HY-14596, MCE), Nystatin (cat# HY-17409, MCE), and Filipin (cat# F4767, MilliporeSigma); macropinocytosis inhibitors EIPA (cat# A3085, MilliporeSigma, Burlington, MA, United States) and LY294002(cat# HY-10108, MCE); and Dynasore (cat# D7693, MilliporeSigma, Burlington, MA, United States) to inhibit both ClME and CaME. As positive controls, pHrod red-transferrin (cat# P35376, Thermo Fisher Scientific, Waltham, MA, United States), Alexa Fluor 488-cholera toxin subunit B (cat# C34775, Thermo Fisher Scientific, Waltham, MA, United States), and Oregon Green 488-dextran (70KD, cat# D7172, Thermo Fisher Scientific, Waltham, MA, United States) were used to confirm the potency of the above inhibitors. Cholesterol absorption inhibitor ezetimibe (cat# HY-17376, MCE), a competitive inhibitor of the lysosomal acid lipase lalistat 2 (cat# SML2053, MilliporeSigma, Burlington, MA, United States), lysosomotropic agents including bafilomycin-A1 (Baf-A1) (cat# HY-100558, MCE), NH_4_Cl, and chloroquine (ChQ) (cat# PHR1258, MilliporeSigma, Burlington, MA, United States) were also used to test the internalization pathway of EVs. All test reagents were used at concentrations that were pre-determined to have no cytotoxicity on HSC or hepatocytes; the concentrations used for each reagent are shown in the figure legends. The cells were then shifted to 37°C to initiate the EV uptake process. Echistatin, a potent inhibitor or RGD-binding integrins (cat#E1518, MilliporeSigma, Burlington, MA, United States), or heparin (cat#H4784, MilliporeSigma, Burlington, MA, United States) were used to test the involvement of cell surface integrin or heparin-like molecules in mediating EV uptake. For this, the recipient cells were pretreated with or without echistatin while EVs were pretreated with or without heparin, at 37°C for 1 h before EV incubation with the recipient cells in the presence of echistatin or heparin alone or in combination. At 24 h post-EV addition, the cells were washed extensively with PBS to remove unbound EVs, fixed with 4% paraformaldehyde, counterstained with DAPI, and photographed with an LSM 800 microscope (Carl Zeiss Inc., Thornwood, NY, United States). PKH26 or RNAselect fluorescence intensity (EV uptake) was quantified using ImageJ (NIH, Bethesda, MD). Alternatively, the cells were lysed at the end of the EV uptake assay and a spectrophotometer (Spectra Max M2, VWR, Sunnyvale, CA, United States) was used to measure the PKH26 signal at Ex/Em = 540/580 with cut-off = 570 nm.

### Iodixanol Isopycnic Gradient Ultracentrifugation

Purified AML12 cell EVs were loaded on top of an iodixanol cushion (40, 32, 24, 16, and 8%, Serumwerk Bernburg AG, Germany) and ultracentrifuged at 37,500 rpm in a SW55Ti rotor for 17 h at 4°C. Twenty fractions were collected from the top to bottom and the density of each fraction was measured using an Abbe refractometer (Bausch and Lomb, Rochester, NY, United States). FN1 and flotillin-1 in each fraction were detected by Western blot. In some cases, the EVs were pretreated with 10% NP40 at 37 °C for 15 min prior to ultracentrifugation.

### Rate-Zonal Ultracentrifugation

1.5 ml of clarified AML12 cell culture supernatant after low-speed centrifugation or purified EVs from AML12 cells were loaded onto a 10–60% sucrose gradient (0.5 ml each of 60, 40, and 30%; 1 ml of 20%, and 10% sucrose in TNE buffer (10 mM Tris–HCl, pH 8.0, 150 mM NaCl, 2 mM EDTA, kept at 4°C overnight before use), and centrifuged at 42,000 rpm (∼167,000 × g) in a SW55Ti rotor at 4°C for 2 h. Fifteen fractions were manually collected from the top and the distributions of FN1 and flotillin-1 were determined by Western blot.

### CCl_4_-Induced Hepatic Fibrosis in Mice

Wild-type male Swiss Webster mice (4–5 weeks old; *n* = 5 per group) were injected i.p. with CCl_4_ (4 μl in 26 μl olive oil) or corn oil (30 μl) three times per week for 5 weeks as described (Li et al., 2019) using IACUC-approved protocol #04504AR (see above). Some mice received i.p. EV^WT^ or EV^Δ*FN1*^ (3e + 9 particles/dose) three times per week over the last 2 weeks of the experiment according to our published procedures (Li et al., 2019). Mice were sacrificed and individual liver lobes were harvested and snap-frozen for histology measurement and RNA extraction for RT-PCR to detect transcript expression of multiple genes including extracellular matrix (COL1A1, COL3A1, MMP2, and RELN), and cell cycle (CCNB2, CDC25C, and KIF2C).

### Histology

Perfused mouse livers were fixed with 4% paraformaldehyde and embedded in paraffin. Sections with 5 μm thickness were cut and stained with H&E. Sections were stained with 0.1% Sirius Red (MilliporeSigma) for collagen detection. Positive signals were quantified by ImageJ analysis.

### RNA Extraction and RT-qPCR

Total RNA from liver tissues or cultured cells was extracted using a miRNeasy mini kit (Qiagen, Germantown, MD, United States) and reverse transcribed with a miScript II RT kit (Qiagen) according to the manufacturer’s instructions. Transcript expression was evaluated by qPCR using SYBR Green Master Mix (Eppendorf, Enfield, CT, United States) on an Eppendorf Mastercycler System. Primers are shown in Table 1. Each reaction was run in duplicate, and samples were normalized to 18S rRNA.

**TABLE 1:** Primers for qRT-PCR.

Gene ID	Accession No.	Primer		Length (bp)
		
		Fwd Seq (5′-3′)	Rev Seq (5′-3′)	
Col3a1	NM_009930	GCCCACAGCCTTCTACACCT	GCCAGGGTCACCATTTCTC	110
MMP2	NM_008610	GCAGCTGTACAGACACTGGT	ACAGCTGTTGTAGGAGGTGC	182
Reln	MMU24703	TTACTCGCACCTTGCTGAAAT	CAGTTGCTGGTAGGAGTCAAAG	73
CCN2	NM_010217	CACTCTGCCAGTGGAGTTCA	AAGATGTCATTGTCCCCAGG	111
Col1a1	NM_007742	GCCCGAACCCCAAGGAAAAGAAGC	CTGGGAGGCCTCGGTGGACATTAG	148
αSMA	NM_007392	GGCTCTGGGCTCTGTAAGG	CTCTTGCTCTGGGCTTCATC	148
CCNB1	NM_172301	AAGGTGCCTGTGTGTGAACC	GTCAGCCCCATCATCTGCG	228
cdc25c	NM_009860	ATGTCTACAGGACCTATCCCAC	ACCTAAAACTGGGTGCTGAAAC	67
KIF2C	NM_134471	ATGGAGTCGCTTCACGCAC	CCACCGAAACACAGGATTTCTC	121
18S	X03205	GGTGAAATTCTTGGACCGGC	GACTTTGGTTTCCCGGAAGC	196

### Statistical Analysis

Experiments were performed at least twice in duplicate or triplicate, with data expressed as mean ± SEM. Fluorescence images were scanned and quantified using ImageJ software (NIH). Data from qRT-PCR and imaging were analyzed by student’s *t*-test. *P-*values < 0.05 were considered statistically significant.

## Results

### Proteomic Analysis of EVs From AML12 Cells

**FIGURE 1:**
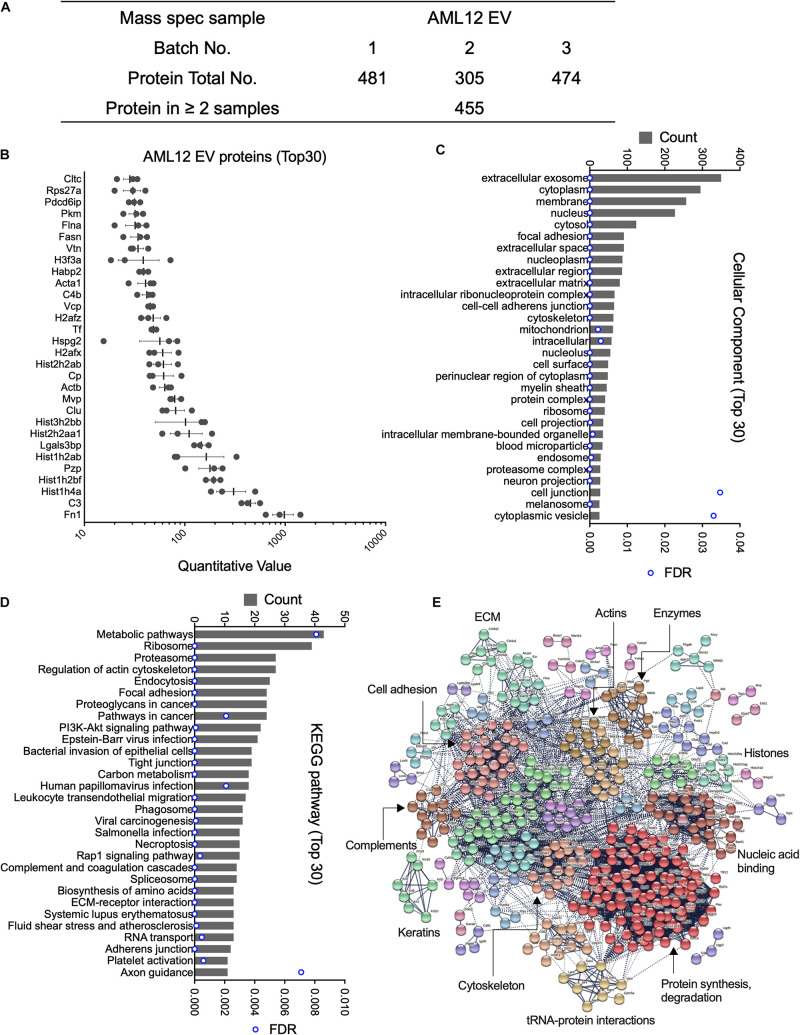
Proteomic analysis of EVs from AML12 cells. **(A)** Summary of quantitative features of EV proteins analyzed from AML12 cell EV samples. **(B)** The 30 most abundant proteins identified in AML12 cell EVs. **(C)** The 30 most enriched components identified by cellular component analysis of all EV proteins. **(D)** The top 30 pathways (from a total of 61 pathways) identified by KEGG pathway analysis of all EV proteins. **(E)** String analysis for entire proteome in AML12 cell EVs, important clusters were labeled.

Mass spectrometry analysis of three separate AML12 cell EV samples resulted in the identification of 481, 305, and 474 proteins, respectively (Figure 1A and Supplementary Table 1). Subsequent analysis was focused on 455 proteins that were present in at least two EV samples. Of these, the most abundant proteins (quantitative value ∼100–1000) in AML12 cell EVs included FN1 (quantitative value ∼1000), complement 3, histones (Hist1h4a, Hist1h2bf, Hist1h2ab, Hist1h2aa1, and Hist3h2bb), pregnancy-zone protein (PZP), galectin 3 binding protein (LGALS3BP), Clu, and MVP (Figure 1B). The identification of Clu and MVP is consistent with their presence and function in EVs from other systems (Foglio et al., 2015; Teng et al., 2017). When GO analysis was used to group all 455 EV proteins into cell components, the EV proteome was characterized as being highly enriched for components related to extracellular exosomes (350 out of 455; 76.9%) (Figure 1C and Supplementary Table 2). Other enriched components included cytoplasm (295), membrane (257), nucleus (227), cytosol (124), focal adhesion (91) and extracellular space (91) (Figure 1C). KEGG pathway analysis revealed 61 enriched pathways, for which metabolic pathways, ribosome, proteasome, regulation of actin cytoskeleton, and endocytosis were ranked as the top five pathways (Figure 1D). STRING analysis of the proteomic data resulted in a complex interaction network, in which principal nodes contained proteins associated with protein synthesis and degradation, nucleic acid binding, histones, enzymes, actins, ECM, cell adhesion, complements, keratins, cytoskeletons, and tRNA-protein interactions (Figure 1E).

### FN1 Is Associated With Hepatocyte EVs

**FIGURE 2:**
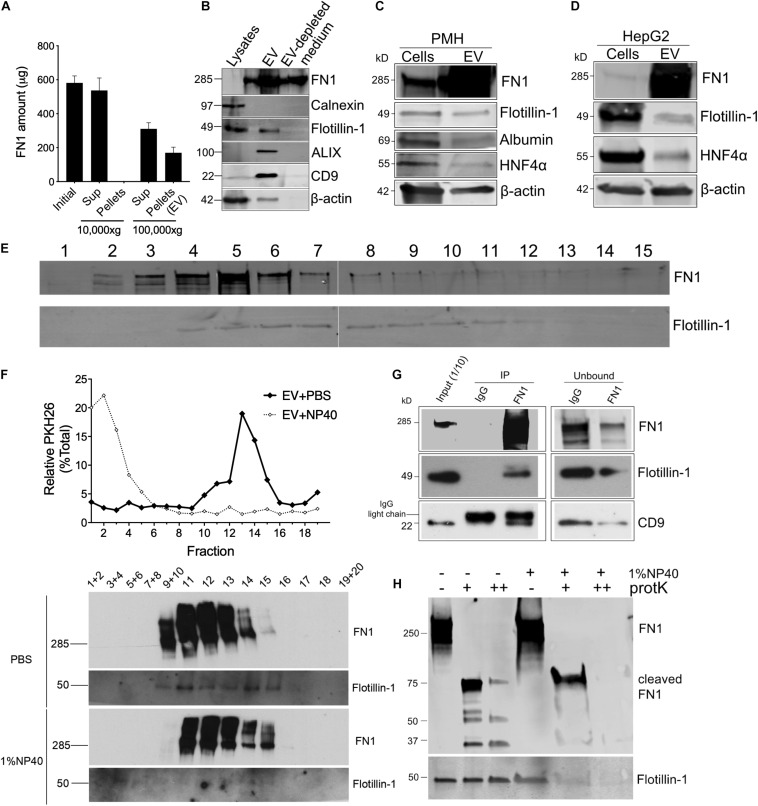
FN1 is associated with hepatocyte-derived EVs. **(A)** Detection by ELISA of FN1 in fractions of AML12 cell conditioned medium after differential centrifugation. **(B)** Western blot detection of FN1, cellular or EV markers in cell lysates, EVs purified from conditioned medium, or EV-depleted medium from AML12 cells. Western blot detection of FN1 and EV markers in cell lysates of and EVs derived from **(C)** primary mouse hepatocytes (PMH) or **(D)** human HepG2 hepatoma cells. 20 μg of total protein was loaded in each lane. **(E)** Distribution of FN1 or flotillin-1 after rate zonal sucrose ultracentrifugation of EVs purified from AML12 cells. **(F)** PKH26-labeled AML12 cell EVs treated with or without 1% NP40 were subjected to iodixanol gradient ultracentrifugation and the resultant fractions were tested for PKH26 by spectrometry (upper panel) and for FN1 or flotillin-1 by Western blot (lower panel). **(G)** FN1 or isotype IgG control was used for immunoprecipitation of AML12 cell EV^WT^, followed by detection of FN1 and EV markers of the pull down material or the unbound fraction. **(H)** AML12 cell EV^WT^ treated with (+) or without (–) 1% NP40 was digested with proteinase K (+20 μg/ml, ++200 μg/ml) after which FN1 and flotillin-1 were detected by Western blot. The experiments were repeated at least 2 times in duplicate.

Proteomic analysis of EV^WT^ showed that FN1 ranked as the most abundant protein (Figure 1B). To validate the presence of FN1 in hepatocyte EVs, conditioned medium from mouse hepatocyte AML12 cells was subjected to differential centrifugation and the distribution of FN1 in each fraction was measured by ELISA. As shown in Figure 2A, high speed (10, 000 × *g*) centrifugation did not result in FN1 loss in the supernatants, and no FN1 was detected in the pellets after high-speed centrifugation. By contrast, approximately two-thirds of FN1 were present in the supernatant after ultracentriguation (100,000 × *g*) while the remaining one-third of FN1 was precipitated with EV pellets. Western blot was subsequently used to demonstrate the presence of FN1 in purified preparations of EV^WT^ which were also positive for EV marker proteins such as flotillin-1, ALIX, and CD9 but negative for the cell-specific marker Calnexin (Figure 2B). FN1 was barely detected in AML12 cell lysates but it was still present in EV-depleted conditioned medium consistent with its release, in part, from the cells as a soluble (non-EV) component (Figure 2B). The structure of EV FN1 was determined from three separate mass spectrometry sequencing analyses in which 1648 to 1760 of the 2477 amino acids in the primary protein sequence (67–71% coverage) were individually identified. Interestingly, the EDB domain was not detected but 26 to 39 amino acids of the 88-residue EDA domain were detected, albeit at a lower coverage rate (29.5–44.3%) than for full-length FN1 (Supplementary Figure 1). To confirm that the FN1 data were not limited to EV^WT^ from the AML12 mouse hepatocyte line, similar EV preparations were purified from primary mouse hepatocytes or the human hepatocyte HepG2 cell line with the result that FN1 was associated with EVs that were also positive for hepatocyte markers (albumin, HNF-4α) and EV markers (flotillin-1) (Figures 2C,D). We have previously shown that HepG2 EVs are also positive for other EV markers including Alix, CD9, and Tsg101 (Li et al., 2019). Thus, FN1-associated EVs were broadly produced by primary or immortalized hepatocytes of human or mice origin.

The results above suggested that while FN1 was present in hepatocyte conditioned medium in its free form which is consistent with its known properties as a secreted protein, an appreciable quantity of FN1 was also EV-associated. This latter possibility was supported by the observation that when purified EVs were subjected to rate zonal ultracentrifugation, the peak of FN1 immunoreactivity (fractions #4–6) was coincident with the presence of flotillin-1 in the same fractions (Figure 2E). Similarly, iodixanol isopycnic ultracentrifugation of EV^WT^ that had been labeled with PKH26 membrane dye resulted in co-distribution of the signals for PKH26 or flotillin-1 (Figure 2F). Moreover, destruction of EV structural integrity using NP40 detergent resulted in liberation of free PKH26, disappearance of the flotillin-1 signal, and a concomitant change in FN1 density shown by a shift to the right of the FN1 signal (Figure 2F). The FN1-association with EVs was further supported by the detection of flotillin-1 or CD9 in anti-FN1 immunoprecipitation of purified EVs, a result that was accompanied by correspondingly diminished intensities of the FN1, flotillin-1 and CD9 signals in the residual unbound sample after immunoprecipitation (Figure 2G). Finally, proteinase K digestion dose-dependently degraded FN1 in EV^WT^ preparations resulting in the production of variably sized fragments (75, 50, 37 kDa) that were resistant to further breakdown (Figure 2H). However, pre-treatment of EV^WT^ with NP40 resulted in complete digestion of FN1 showing the importance of EV structural integrity for protecting FN1 from proteolysis, as was also observed for flotillin-1 (Figure 2H). The susceptibility of FN1 to digestion by proteinase K in the absence of NP40 suggests that FN1 is associated peripherally, likely on the EV surface, which is fully consistent with the role for FN1 in mediating EV binding to target cells (see below). Since NP40 facilitates proteinase K digestion of FN1, this likely reflects the partial association of FN1 with the EV membrane, possibly by direct anchoring or by being tethered to a binding partner within the EV membrane. In contrast, flotillin-1 is a fully membrane-associated protein (Otto and Nichols, 2011) accounting for its proteinase K resistance unless the membrane is disrupted by NP40. Overall, these various approaches lend strong support for an intimate association between FN1 and hepatocyte EVs.

### Generation of FN1-Deficient Cells and EVs

**FIGURE 3:**
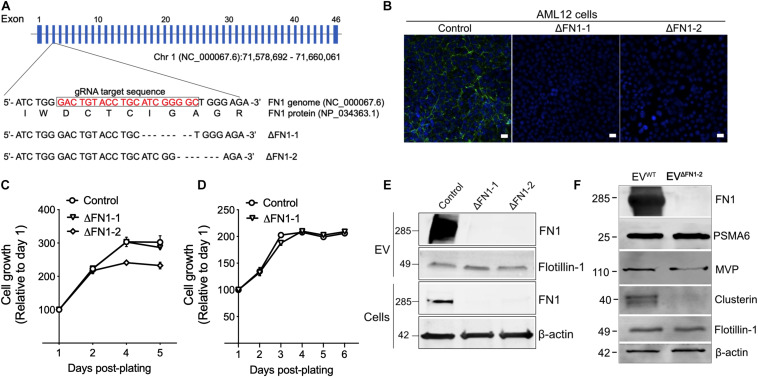
Generation of ΔFN1 AML12 cells. **(A)** mFN1 genomic structure showing the gRNA target sequence on exon 3. Also shown is the result of genomic sequencing of two knockouts. **(B)** Immunofluorescence detection of total FN1 with the specific antibody (in green) in WT AML12 cells but not in ΔFN1-1 or ΔFN1-2 AML12 cells. Scale bar = 50 μm. Growth curve of WT or ΔFN1 cells in **(C)** 10% FBS-containing medium or **(D)** serum-free medium. **(E)** Western blots showing FN1 or flotillin-1 signals in EV^WT^, EV^Δ*FN1*– 1^, or EV^Δ*FN1*– 2^ (*upper two panels*) and FN1 or β-actin in WT, ΔFN1-1, or ΔFN1-2 cells (*lower two panels*); 10 μg of proteins were loaded each lane. **(F)** Expression comparison of EV proteins in EV^WT^ or EV^Δ*FN1*– 2^; 20 μg of total EV protein (approximately 2.5E + 9 particles) was loaded in each lane. PSMA6, MVP, and clusterin were identified in the proteome of EVs from AML12 cells (see Figure 1B). The experiments were repeated at least 2 times in duplicate.

To investigate the biological function of EV-associated FN1, ΔFN1 AML12 cells were first generated using CRISPR-Cas9. Genome sequencing (Figure 3A) and immunofluorescence assay (Figure 3B) both confirmed the knockout of FN1 in two single clones (ΔFN1-1 and ΔFN1-2). As assessed using alamarBlue reagent, cell growth kinetics under normal (Figure 3C) or serum-free condition (Figure 3D) were not significantly affected by FN1 deficit. When purified EVs were assessed by Western blot, no FN1 was detected in EV^Δ*FN1*^ unlike the substantial FN1 signal in EV^WT^ (Figure 3E). Interestingly, MVP and Clu (Figure 1B) were present in EV^Δ*FN1*^ at highly reduced levels as compared to EV^WT^ whereas the signal for proteasome subunit alpha type-6 (PSMA6) and flotillin-1 was comparable between the two types of EVs (Figure 3F).

### FN1 Deficit Reduces EV Uptake

**FIGURE 4:**
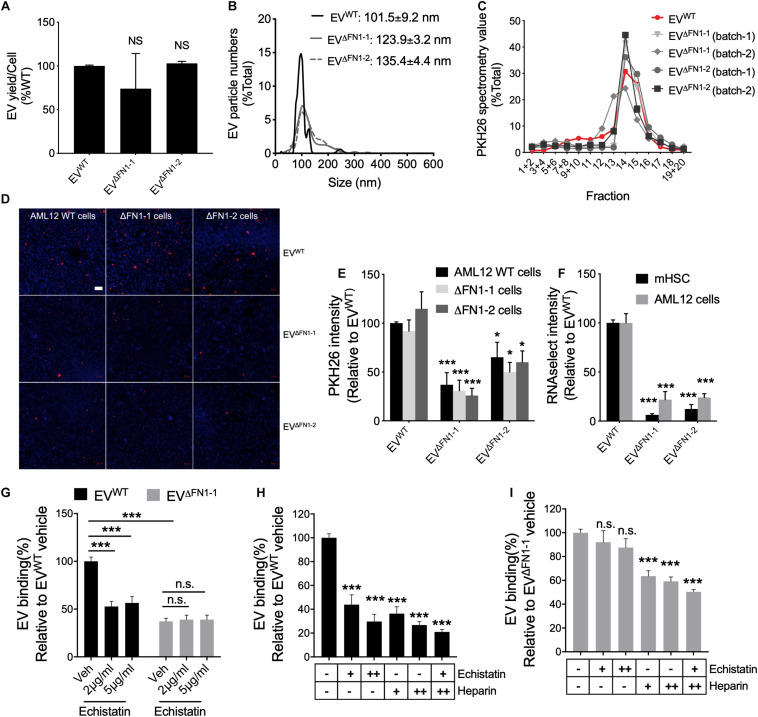
FN1 is not required for EV biogenesis but facilitates EV uptake. **(A)** EV yield from 48 h-conditioned medium of WT, ΔFN1-1 or ΔFN1-2 cells. **(B)** Size distribution and frequency of EV^WT^, EV^Δ*FN1*– 1^, or EV^Δ*FN1*– 2^ measured by NTA. **(C)** Density profile of PKH26-labeled EV^WT^, EV^Δ*FN1*– 1^, or EV^Δ*FN1*– 2^ after isopycnic iodixanol gradient ultracentrifugation. **(D)** Representative images showing uptake of PKH26-labeled EV^WT^, EV^Δ*FN1*– 1^, or EV^Δ*FN1*– 2^ after incubating WT, ΔFN1-1 or ΔFN1-2 AML12 cells with 1e + 9 EVs/ml for 24 h. Scale bar = 50 μm. **(E)** Quantification of data in (D) based on counting 2 fields for each of the three independent experiments. **(F)** Uptake of RNAselect-labeled EV^WT^, EV^Δ*FN1*– 1^, or EV^Δ*FN1*– 2^ in primary activated mHSC or AML12 cells that were incubated with 2e + 8 EVs/ml for 24 h. **(G)** Uptake of PKH26-labeled EV^WT^ or EV^Δ*FN1*– 1^ by primary activated mHSC that were incubated with EVs containing the same PKH26 signal for 24 h in the presence of 0–5 μg/ml echistatin before measurement of PKH26 intensity by spectrometry. Uptake of **(H)** EV^WT^ or **(I)** EV^Δ*FN1*– 1^ by primary activated mHSC that were incubated with 2e + 8 EVs/ml for 24 h in the presence of echistatin (+, 2 μg/ml; ++, 5 μg/ml) and/or heparin (+, 50 μg/ml; ++, 100 μg/ml). The experiments were repeated at least 2 times in duplicate. *P < 0.5, ***P < 0.005.

To evaluate the consequences of FN1 deficit, the EV yield was firstly assessed. WT or ΔFN1 AML12 cells were incubated with serum-free medium for 48 h, followed by EV purification and quantification by NTA. The EV yield was calculated by EV numbers/cell and normalized to WT. As shown in Figure 4A, no significant alterations were detected in either ΔFN1-1 or ΔFN1-2 cells. NTA showed that while the overall profiles of EV^Δ*FN1*^ were similar to that of EV^WT^, the average sizes of EV^Δ*FN1*–1^ (123.9 ± 3.2 nm) or EV^Δ*FN1*–2^ (135.4 ± 4.4 nm) were approximately 10–20% greater than EV^WT^ (101.5 ± 9.2 nm) (Figure 4B). A higher percentage of EV^Δ*FN1*^ with larger size (≥120 nm) was seen compared to EV^WT^ (Figure 4B) but it remains to be determined if this size difference represents differences in pathways of EV biogenesis or a structural contribution by FN1 to EV ‘compactness.” That said, the buoyant density of the EVs was unaffected by FN1 knockout as shown by the sedimentation of EV^WT^, EV^Δ*FN1*–1^, or EV^Δ*FN1*–2^ over the same density range (fractions 12–16) upon iodixanol isopycnic ultracentrifugation (Figure 4C). To assess the efficiency of EV cellular uptake of the EVs after FN1 knockout, equivalent numbers of EV^WT^, EV^Δ*FN1*–1^, or EV^Δ*FN1*–2^, all with comparable PKH26 signal, were incubated with WT or ΔFN1 AML12 cells. EV^WT^ uptake efficiency was comparable in WT or ΔFN1 cells, suggesting that cell-associated FN1 is dispensable for EV uptake (Figures 4D,E). By contrast, uptake of EV^Δ*FN1*–1^ or EV^Δ*FN1*–2^ to WT or ΔFN1 cells was reduced by 50–70% of control values (Figures 4D,E), showing that EV-associated FN1 facilitates (but is not essential for) EV uptake. As compared to EV^WT^, EV^Δ*FN1*^ labeled with RNAselect produced a weaker signal in mHSC or AML12 (Figure 4F) showing that EV-mediated RNA delivery was dependent on EV FN1. Echistatin, a potent inhibitor of RGD-binding integrins, significantly inhibited cellular uptake of EV^WT^ but not of EV^Δ*FN1*^ (Figure 4G), showing that integrin-FN1 interactions are important for EV^WT^ uptake. Treatment with soluble heparin also dose-dependently inhibited cellular binding of either EV^WT^ (Figure 4H) or EV^Δ*FN1*^ (Figure 4I) but the heparin-mediated inhibition was less robust for EV^ΔFN1^.

### Involvement of Endocytosis and Macropinocytosis in EV Uptake

**FIGURE 5:**
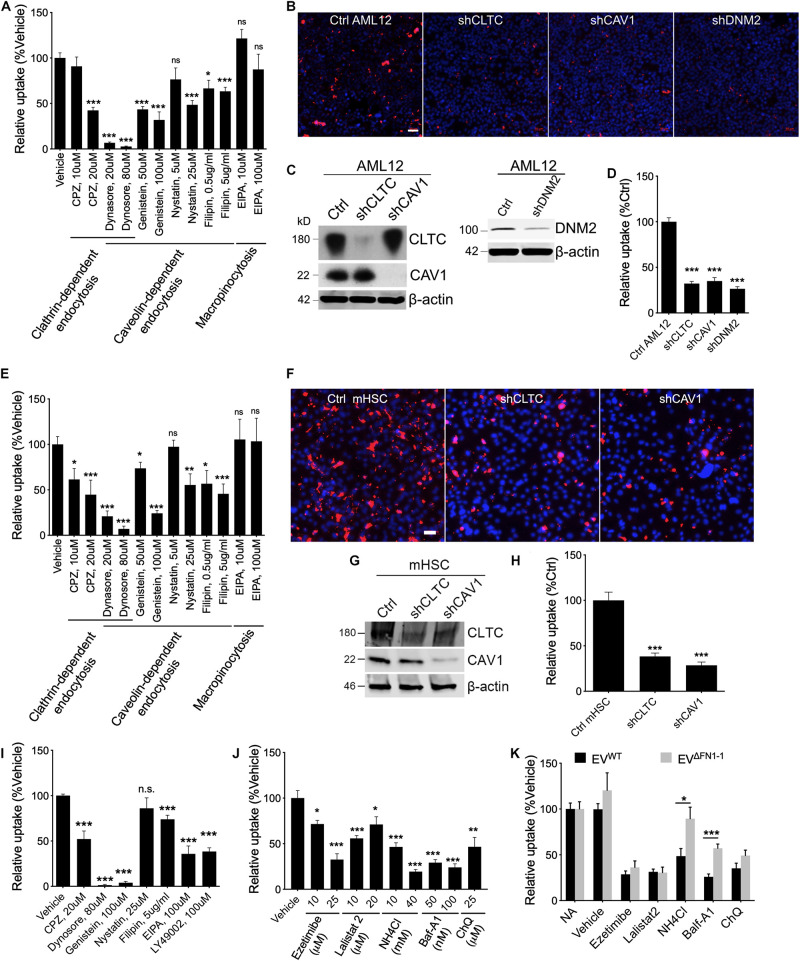
Involvement of endocytosis, macropinocytosis, lysosome in EV^WT^ or EV^Δ*FN1*^ uptake. **(A)** Uptake of PKH26-labeled EV^WT^ by AML12 cells incubated in the presence of 2e + 9 EVs/ml for 24 h and inhibitors of endocytosis or macropinocytosis. **(B)** Representative images of uptake of PKH26-labeled EV^WT^ by WT AML12 cells that were incubated with 2e + 9 EVs/ml for 3 h or by AML12 cells that expressed reduced levels of clathrin heavy chain (CLTC), caveolin-1 (CAV1), or Dynamin-2 (DNM2). Scale bar = 50 μm. **(C)** Western blot detection of CLTC or CAV1 or DNM2 in AML12 cells transduced with lentiviral shCLTC, shCAV1, or shDNM2. **(D)** Quantification of data shown in **(B)** from analysis of 2–9 fields/independent experiment. **(E)** Uptake of PKH26-labeled EV^WT^ by primary activated mHSC incubated in the presence of 2e + 9 EVs/ml for 24 h and inhibitors of endocytosis or macropinocytosis. **(F)** Representative images of uptake of PKH26-labeled EV^WT^ by primary activated mHSC that were incubated with 2e + 9 EVs/ml for 3 h or by mHSC that expressed reduced levels of CLTC or CAV1. Scale bar = 50 μm. **(G)** Western blot detection of CLTC or CAV1 in mHSC transduced with lentiviral sh CLTC or shCAV1. **(H)** Quantification of data shown in (B) from analysis of 2–4 fields/independent experiment. **(I)** Uptake of PKH26-labeled EV^Δ*FN1*– 1^ by primary activated mHSC that were incubated with 2e + 9 EVs/ml for 24 h and treated with inhibitors of endocytosis or macropinocytosis. **(J)** Effect of different concentrations of Ezetimibe, Lalistat 2, NH_4_Cl, Bafilomycin A1 (Baf-A1), or chloroquine (ChQ) on uptake of PKH26-labeled EV^WT^ by activated primary mHSC that were incubated with 2e + 9 EVs/ml for 24 h. **(K)** Comparison of uptake of EV^WT^ or EV^Δ*FN1*– 1^ by activated primary mHSC treated with Ezetimibe, Lalistat 2, NH4Cl, Baf-A1, or ChQ. The experiments were repeated at least 2 times in duplicate. *P < 0.5, **P < 0.01, ***P < 0.005.

To investigate which pathways are involved in EV uptake, a panel of inhibitors to ClME, CaME, and macropinocytosis were used for a small-scale screening. First, the cell cytotoxicity (Supplementary Figure 2) and the ability of the reagents to inhibit endocytosis or macropinocytosis was validated using, respectively, fluorophore-labeled transferrin (for ClME), Cholera enterotoxin subunit B (CtxB, for CaME), or dextran (for macropinocytosis) (Supplementary Figure 3). Next, EV^WT^ were added to WT AML12 cells pretreated with different reagents for 1 h and the EVs and reagents were then simultaneously incubated with the cells for another 24 h. Inhibitors of endocytosis but not of macropinocytosis reduced the uptake of EV^WT^ in a dose-dependent manner, with dynasore having the most potent inhibition (Figure 5A). Whereas uptake of PKH26-labeled EV^WT^ by WT AML12 cells was readily visualized by the presence of PKH26 fluorescence in the cells (Figure 5B), shRNA-mediated knockdown of CLTC, CAV1, or DNM2, which resulted in a significant decrease in expression of each component (Figures 5C,D), caused the cellular binding of EV^WT^ be impaired by more than 50% as compared to WT cells (Figures 5B,D). Similarly, EV^WT^ uptake by mHSC was reduced by inhibitors of clathrin- or caveolin-mediated endocytosis, but not of macropinocytosis (Figure 5E) and the fluorescent signal associated with the uptake of PKH26-labeled EV^WT^ by mHSC was reduced by prior knockdown of CLTC or CAV1 in the mHSC target cells (Figures 5F–H).

To understand if EV^Δ*FN1*^ utilized the same mechanisms to enter cells, mHSC were pretreated with the endocytosis inhibitors and incubated with EV^Δ*FN1*^ in the presence of the inhibitors for 24 h before imaging and quantification. Clathrin- or caveolin-mediated endocytosis was shown to be required for EV^Δ*FN1*^ uptake, as the inhibitors to either pathway significantly reduced the uptake (Figure 5I). Interestingly, two macropinocytosis inhibitors (EIPA and LY49002) individually inhibited the EV^Δ*FN1*^ uptake (Figure 5I), showing EV^Δ*FN1*^ can enter cells through macropinocytosis pathway even though EV^WT^ did not (Figures 5A,E). Similar with EV^WT^, shRNA-mediated knockdown of CLTC or CAV1 significantly reduced EV^Δ*FN1*^ uptake in mHSC (Supplementary Figure 4).

To evaluate if cholesterol, lysosomal acid lipase, or lysosomal pH are involved in EV uptake, mHSC were pretreated with cholesterol absorption antagonist ezetimibe (Chang and Chang, 2008), lysosomal acid lipase inhibitor lalistat 2 (Hamilton et al., 2012), or lysosomotropic agents including Baf-A1, NH_4_Cl, or ChQ before EV inoculation. The uptake of EV^WT^ by mHSC was susceptible to each of these agents (Figure 5J), suggesting the involvement of cholesterol and lysosome, in which acid lipase and the low pH conditions are both required for the following possible membrane fusion step. By contrast, uptake of EV^Δ*FN1*^ was also sensitive to ezetimibe or lalistat 2 treatment but more resistant to lysosomotropic agents (NH_4_Cl, Baf-A1) (Figure 5K) suggesting that FN1 may facilitate low pH-mediated EV entry.

### FN1 Deficient EVs Are Still Therapeutic for Experimental Liver Fibrosis

**FIGURE 6:**
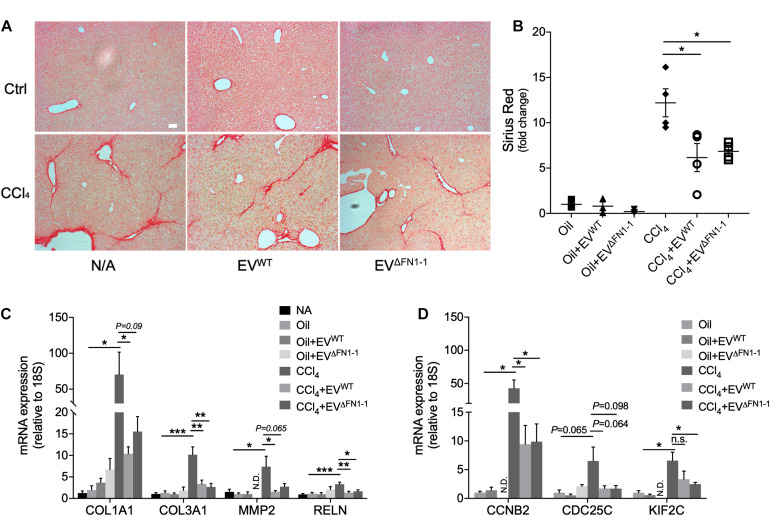
FN1 deficit does not affect the therapeutic effect of hepatocyte EVs in experimental fibrosis *in vivo*. **(A)** Sirius red staining of collagen deposition in liver tissues from Swiss Webster mice (5 male mice per group) that were treated with oil (CCl_4_ carrier control) or CCl_4_ for six weeks, some of which also received EV^WT^ or EV^Δ*FN1*– 1^ i.p three times a week over the last 2 weeks. Scale bar = 50 μm. **(B)** Quantification of **(A)** based on 3–4 fields. Quantification by qRT-PCR of **(C)** fibrosis-related genes or **(D)** cell cycle-related genes. The experiments were repeated at least 2 times in duplicate. *P < 0.5, **P < 0.01, ***P < 0.005.

We have previously shown that EV^WT^ are therapeutic for CCl_4_-induced liver fibrosis, resulting in attenuated expression of fibrosis-related and cell cycle related genes (Li et al., 2019). To evaluate if there was a functional difference between EV^Δ*FN1*^ and EV^WT^, each type of EV was administered to mice over the last 2 weeks of a 6-week course of CCl_4_ to induce hepatic fibrosis. CCl_4_-treated mice demonstrated excessive hepatic collagen deposition as compared to control mice (Figure 6A). Administration of either EV^WT^ or EV^Δ*FN1*^ resulted in diminished amounts of collagen deposition as shown by Sirius red staining (Figures 6A,B). The previously reported attenuation by EV^WT^ of CCl_4_-induced extracellular matrix (ECM) genes (COL1A1, COL3A1, MMP2, and RELN) or cell cycle genes (CCNB2, CDC25C, and KIF2C) (Li et al., 2019) was also seen in response to EV^Δ*FN1*^ (Figures 6C,D). Thus EV-associated FN was not required for EV-mediated suppression of collagen deposition or CCl_4_-induced genes.

## Discussion

The principal findings of this study are that FN1 is a major component of hepatocyte EVs that mediates RGD (integrin)- dependent EV binding to target cells and favors EV uptake by endocytic mechanisms that involve low pH and that circumvent macropinocytosis. EV FN1 was dispensable for the interaction of EVs with cell surface heparin-like molecules, for EV uptake by clathrin- and caveolin-mediated endocytosis, and for EV-mediated therapy of CCl_4_-induced hepatic fibrosis in mice.

Our data show that plasma FN1 appeared to be the main form of EV-associated FN1 and this is consistent with plasma FN1 being the principal form secreted by hepatocytes (Tamkun and Hynes, 1983). On the other hand, a 39-residue sequence corresponding to part of the EDA domain was nonetheless detected by mass spectrometry and we cannot therefore rule out the possible presence either of cellular FN1 in which EDA and EDB are difficult to structurally confirm or of a novel FN1 variant containing only a portion of the EDA domain. A prior study has reported the presence of EDA-containing FN1 in MSC-derived EVs (Lai et al., 2016). Our analysis of several hepatocyte cell types from two different species (human, mouse) showed that FN1 consistently existed in EV-associated forms. EV-associated FN1 was characterized as such by the presence of EV components in FN1 immunoprecipitates, its co-sedimentation with EV markers, and its resistance to proteinase K, the latter two of which were dependent on EV membrane integrity. In addition, the lack of FN1 in EVs produced by ΔFN1 cells and the differences in cellular uptake and trafficking of EV^Δ*FN1*^ versus EV^WT^ provide strong support for FN1 as a *de facto* component of EVs.

EV^Δ*FN1*^ were taken up by hepatocytes less efficiently than EV^WT^, indicating that FN1 is a ligand for EV binding to recipient cells. This is consistent with our prior report (Chen and Brigstock, 2016) that hepatocyte EVs interact with cell surface integrin αvβ3, integrin α5β1, and HSPGs, all of which are FN1-binding moieties albeit with distinct patterns of interaction (Kennelly et al., 2019). However, while the binding of EV^Δ*FN1*^ to hepatocytes was resistant to displacement by echistatin, it was still susceptible to being blocked by heparin, although the latter occurred to a lesser degree than for EV^WT^. Thus, FN1 was a principal EV ligand for cellular RGD-binding integrins whereas its interaction with cellular HSPGs was partial and shared with other heparin-binding EV components which are as yet unidentified. The reduced sensitivity of EV^Δ*FN1*^ to heparin may reflect the absence in the EVs of FN1 because other studies have shown that FN1 is a heparin-binding protein that acts co-operatively with cellular HSPGs to promote EV binding and uptake by target cells (Purushothaman et al., 2016). Echistatin is a potent antagonist of many RGD-sensitive integrins including αIIbβ3, αvβ3 and α5β1, while FN1 can interact with multiple integrins including αIIbβ3, αvβ1, αvβ3, αvβ6, αvβ8, α4β1, and α5β1. HSPGs are a combination of two or three polysaccharide heparan sulfate chains that are attached in close proximity to transmembrane proteins such as syndecan types 1–4, Glycosylphosphatidylinositol-linked glypicans types 1–6, glycosylphosphatidylinositol, perlecan, agrin, betaglycan, and CD44. Future studies using siRNA, blocking antibodies and other antagonists will be necessary to properly identify receptors on various hepatic cells for EV-associated FN1. Importantly, the anti-fibrotic activities of EV^Δ*FN1*^ were probably comparable to EV^WT^ in long-term (6-week) *in vivo* experiments because the cellular binding and delivery of cargo molecules by EV^Δ*FN1*^ in short-term (24-h) *in vitro* experiments, while less efficient than EV^WT^, was not totally ablated and likely provided for sufficient delivery of a therapeutic cargo over the longer duration. This outcome also showed that FN1 was not itself a therapeutic component of the EV payload.

In other studies, heparan sulfates on myeloma cell-derived EVs were shown to capture FN1 which was then delivered to target cells via its subsequent binding to cell surface heparan sulfate receptors, resulting in activation of p38/pERK and expression of genes that promote myeloma progression (Purushothaman et al., 2016). Transglutaminase and FN1 in cancer cell EVs acted co-operatively to trigger transformation of fibroblast recipient cells, the latter of which engaged the EVs in an integrin-dependent (echistatin-sensitive) manner (Antonyak et al., 2011), while EVs from neural stem cells used cell surface HSPG as receptors prior to being endocytosed by microvascular endothelial cells (Joshi and Zuhorn, 2020). EVs stimulated colony-formation in breast cancer cells after binding to cell surface integrin β3 and undergoing internalization in association with HSPG, with endocytosis being triggered and dependent on activation by EVs of focal adhesion kinase in an integrin-β3-dependent manner (Mulcahy et al., 2014). Finally, FN1 in mesenchymal stem cell-derived EVs was high in a specific EV subtype that could be captured by a GM1 ganglioside-specific ligand CtxB and which contained the majority of the EV RNA payload showing that EV FN1 content is indicative of EV function (Lai et al., 2016).

ClME, CaME, or macropinocytosis are involved in internalization of EVs from different origins (Mulcahy et al., 2014) and we found that hepatocytes and HSC share the same uptake machinery for uptake of EV^WT^, namely CaME and ClME but not macropinocytosis. Interestingly, macropinocytosis was nonetheless involved in the uptake of EV^Δ*FN1*^ in HSC suggesting that FN1 can direct EVs along specific endocytic pathways. Previous reports indicate that FN1 is internalized by CaME and is subsequently degraded in the lysosome by a process that can be inhibited by lysosomotropic agents such as ChQ (Sottile and Chandler, 2005; Shi and Sottile, 2008). In this process, integrins mediate the binding with and the turnover of FN1 suggesting that FN1 may facilitate or mediate EV uptake through the same machinery, as has been demonstrated in cancer cell systems (Antonyak et al., 2011; Fuentes et al., 2020). This may explain why EV^WT^ were more susceptible to lysosomotropic agent treatment as compared to EV^Δ*FN1*^ which, by contrast, did not interact with echstatin-sensitive integrins on target cells and which utilized macropinocytosis for uptake, unlike their EV^WT^ counterparts. Despite the endocytic differences between EV^WT^ and EV^Δ*FN1*^, there was no biological impact, at least in terms of their long-term anti-fibrotic actions *in vivo*. However, other protective aspects of EV action (anti-inflammatory, anti-apoptotic, etc.) might be impacted by the loss of FN1 from EVs and their utilization of different endocytic mechanisms and these possibilities will be explored in future studies. Finally, the differential expression of certain exosome components (MVP, Clu) between EV^WT^ and EV^Δ*FN*1^ supports further proteomic analysis to confirm and extend these initial observations.

## Data Availability Statement

The mass spectrometry proteomics data have been deposited to the ProteomeXchange Consortium via the PRIDE (Perez-Riverol et al., 2019) partner repository with the dataset identifier PXD023860.

## Ethics Statement

The animal studies were reviewed and approved by the Institutional Animal Care and Use Committee of Nationwide Children’s Hospital (Columbus, OH, United States).

## Author Contributions

XL performed the study design, acquisition and analysis of the data, figure preparation, and drafting and editing the manuscript. RC and SK performed the experiment planning, data acquisition, and manuscript editing. DB was responsible for the study concept and design, interpretation of the data, revising the manuscript, funding, and study supervision. All authors contributed to the article and approved the submitted version.

## Conflict of Interest

The authors declare that the research was conducted in the absence of any commercial or financial relationships that could be construed as a potential conflict of interest.

## Footnotes

1 http://geneontology.org/

2 https://www.genome.jp/kegg/

3 https://david.ncifcrf.gov/

4 https://string-db.org/

## Supplementary Material

Supplementary Figure 1 Amino acid sequence data for EV-associated FN1. Mass spectrometry sequencing of FN1 in the **(A)** first, **(B)** second, or **(C)** third AML12 EV samples. Residues that were experimentally determined are shown in yellow highlights. The EDA (blue box) and EDB (red box) domains are arrowed.

Supplementary Figure 2 Cytotoxicity assay of reagents used to block endocytosis, macropinocytosis, or lysosome function. Cytotoxicity of clathrin-mediated endocytosis inhibitors (chlorpromazine, CPZ; Dynasore), caveolin-mediated endocytosis inhibitors (Dynasore, Genistein, Nystain, and Filipin), and macropinocytosis inhibitors (EIPA, LY49002) in **(A)** AML12 cells or **(B)** passaged mHSC. Ezetimibe, lalistat 2, and lysosomotropic agents (NH4Cl, Baf-A1, and ChQ) cytotoxicity in **(C)** AML12 cells or **(D)** passaged mHSC. The experiments were repeated at least 2 times in duplicate.

Supplementary Figure 3 Potency assessments of antagonists to endocytosis or macropinocytosis using fluorophore-labeled molecules. **(A)** Uptake of pHrod red-transferrin in AML12 cells in the context of treatment with clathrin-mediated endocytosis inhibitors, **(B)** Uptake of Alexa Fluor 488-cholera toxin subunit B in AML12 cells in the presence of caveolin-mediated endocytosis inhibitors. **(C)** Uptake of Oregon Green 488-dextran in AML12 cells treated with macropinocytosis inhibitors. The experiments were repeated at least 2 times in duplicate. Scale bar = 50 μm.

Supplementary Figure 4 EV^ΔFN1^ uptake in mHSC with reduced CLTC or CAV1 expression. **(A)** Representative images of uptake of PKH26-labeled EV^Δ*FN1*^ in control, CLTC, or CAV1 knock-down mHSCs that were inoculated with 2e + 9 particle/ml of the EVs for 3 h before cell fixation and imaging. Scale bar = 50 μm. **(B)** Quantification of data shown in **(A)** from analysis of 2 fields/independent experiment.

Supplementary Table 1 Proteome dataset of AML12 cell EV proteins.

Supplementary Table 2 Dataset of 350 proteins in AML12 cell EVs that were classified as extracellular exosomes after by GO analysis of cell components of all EV proteins.
