# Supplementary figures and images for: Structural and Functional Characterization of Fibronectin in Extracellular Vesicles From Hepatocytes
Source: Front Cell Dev Biol. 2021 Mar 18;9:640667. doi: 10.3389/fcell.2021.640667 (PMC8012540; doi:10.3389/fcell.2021.640667)

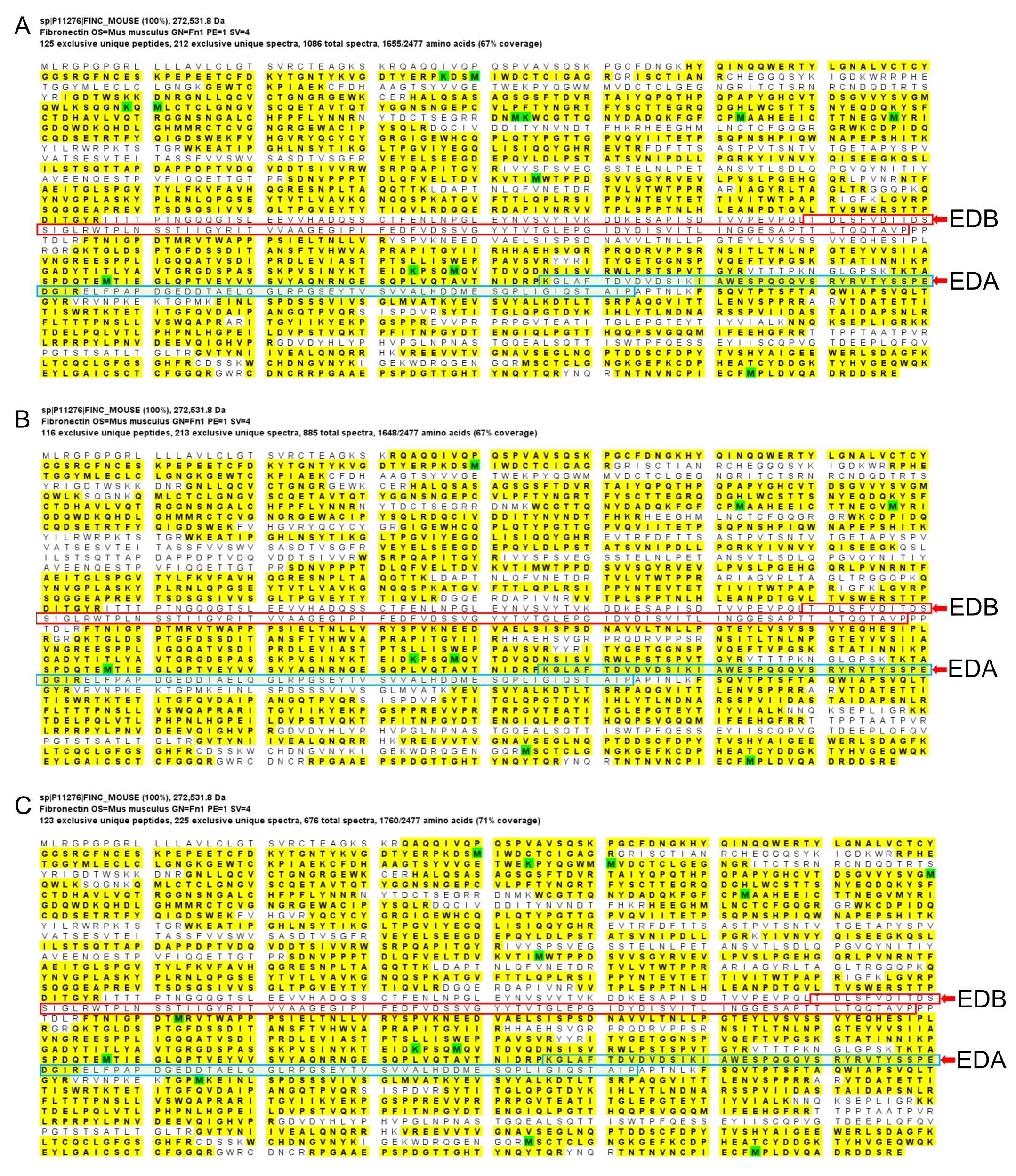

Supplement: Supplementary Figure 1 — Amino acid sequence data for EV-associated FN1. Mass spectrometry sequencing of FN1 in the (A) first, (B) second, or (C) third AML12 EV samples. Residues that were experimentally determined are shown in yellow highlights. The EDA (blue box) and EDB (red box) domains are arrowed. [file Image_1.TIFF]

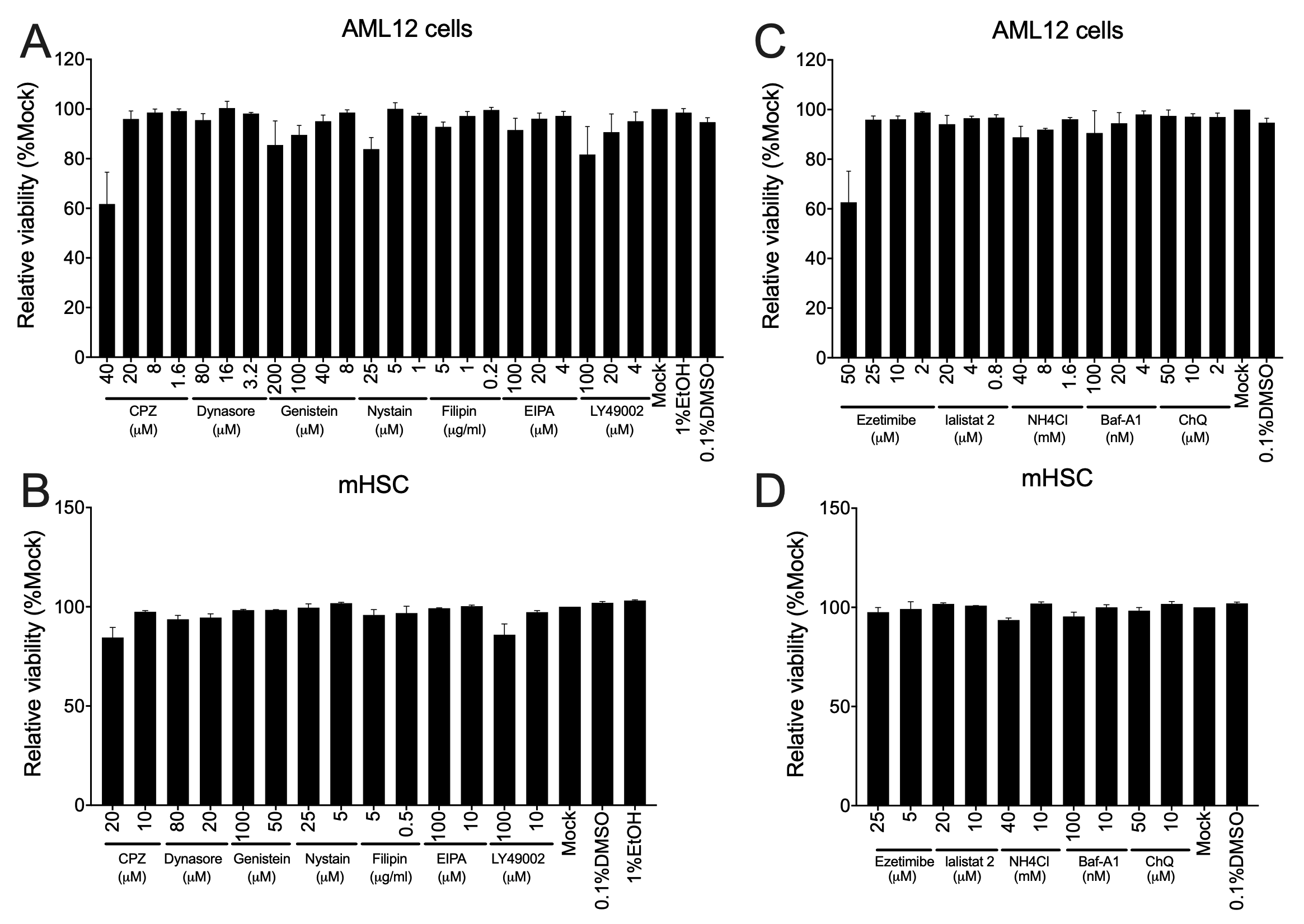

Supplement: Supplementary Figure 2 — Cytotoxicity assay of reagents used to block endocytosis, macropinocytosis, or lysosome function. Cytotoxicity of clathrin-mediated endocytosis inhibitors (chlorpromazine, CPZ; Dynasore), caveolin-mediated endocytosis inhibitors (Dynasore, Genistein, Nystain, and Filipin), and macropinocytosis inhibitors (EIPA, LY49002) in (A) AML12 cells or (B) passaged mHSC. Ezetimibe, lalistat 2, and lysosomotropic agents (NH4Cl, Baf-A1, and ChQ) cytotoxicity in (C) AML12 cells or (D) passaged mHSC. The experiments were repeated at least 2 times in duplicate. [file Image_2.TIFF]

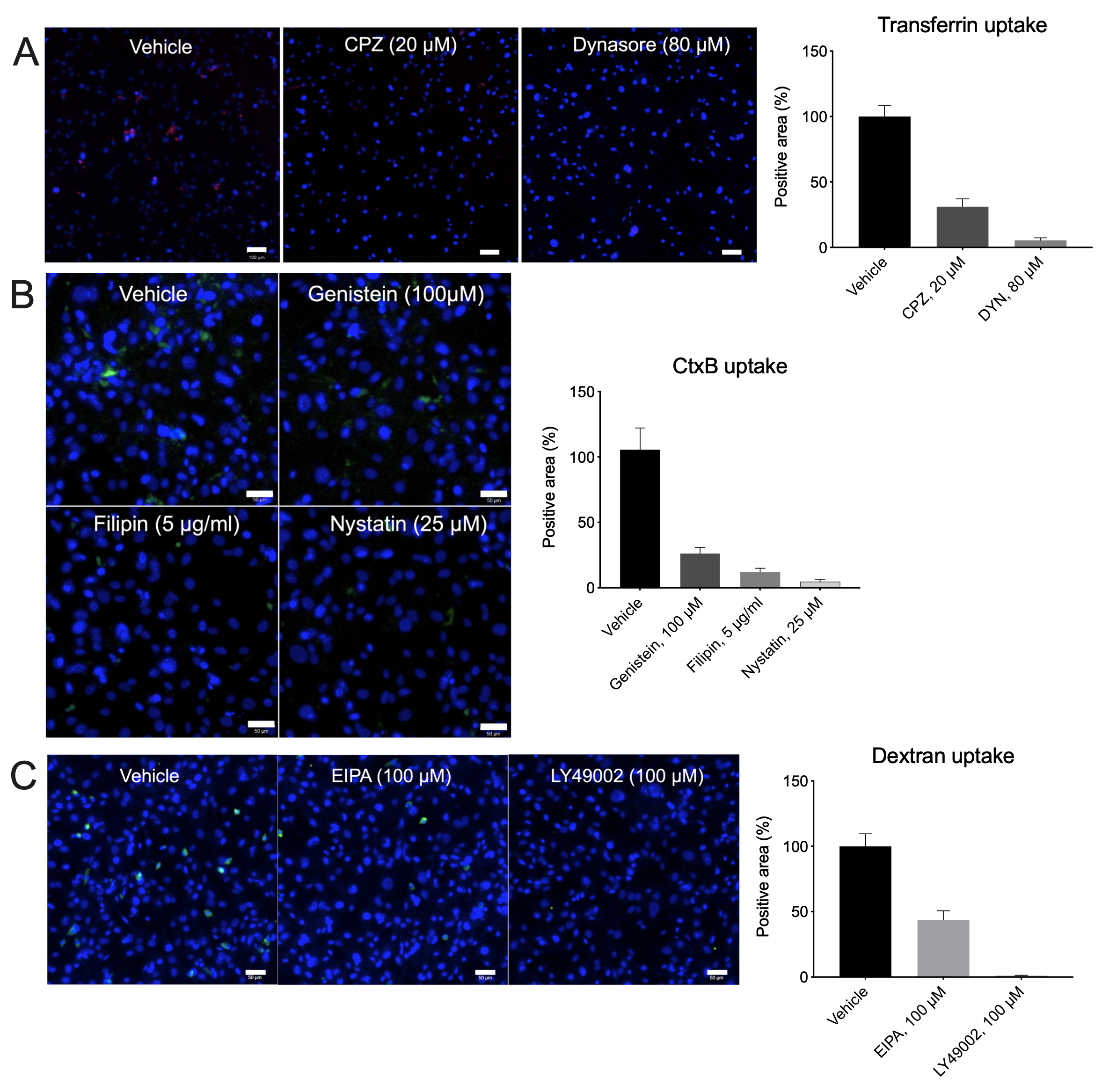

Supplement: Supplementary Figure 3 — Potency assessments of antagonists to endocytosis or macropinocytosis using fluorophore-labeled molecules. (A) Uptake of pHrod red-transferrin in AML12 cells in the context of treatment with clathrin-mediated endocytosis inhibitors, (B) Uptake of Alexa Fluor 488-cholera toxin subunit B in AML12 cells in the presence of caveolin-mediated endocytosis inhibitors. (C) Uptake of Oregon Green 488-dextran in AML12 cells treated with macropinocytosis inhibitors. The experiments were repeated at least 2 times in duplicate. Scale bar = 50 μm. [file Image_3.TIFF]

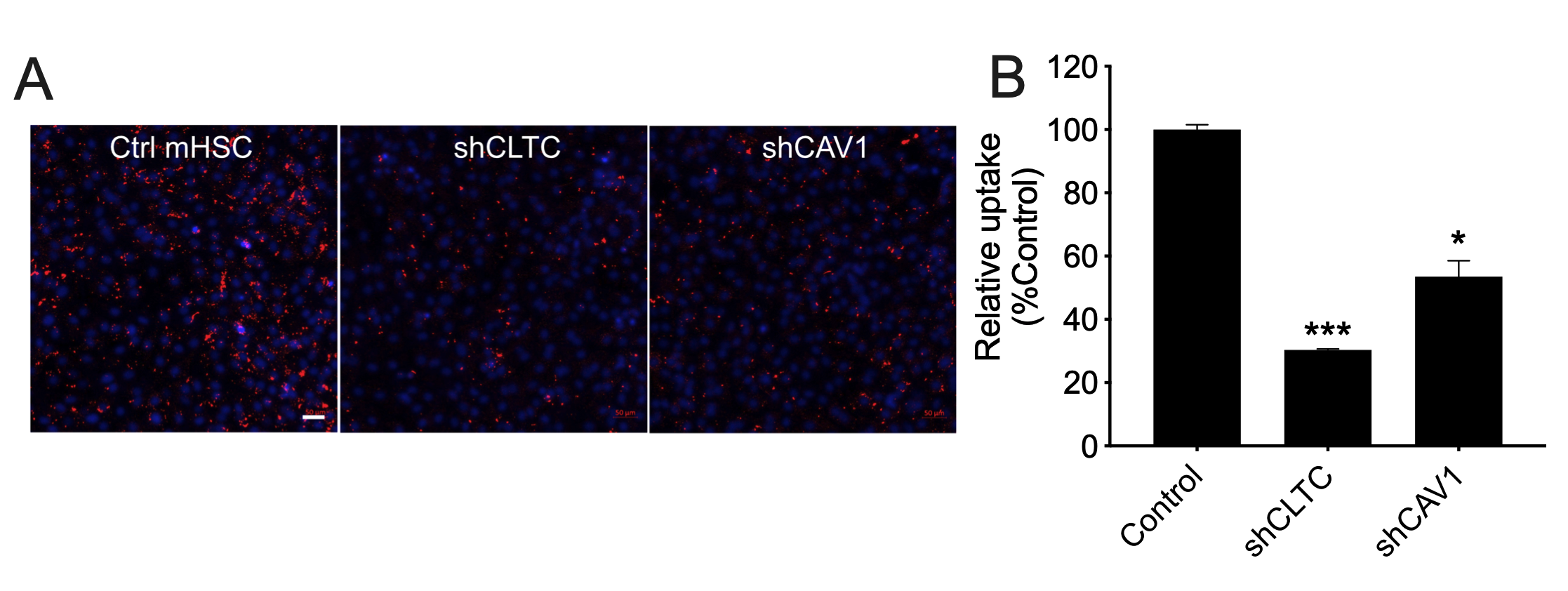

Supplement: Supplementary Figure 4 — EVΔFN1 uptake in mHSC with reduced CLTC or CAV1 expression. (A) Representative images of uptake of PKH26-labeled EVΔFN1 in control, CLTC, or CAV1 knock-down mHSCs that were inoculated with 2e + 9 particle/ml of the EVs for 3 h before cell fixation and imaging. Scale bar = 50 μm. (B) Quantification of data shown in (A) from analysis of 2 fields/independent experiment. [file Image_4.TIFF]
